# Phimosis with Exaggerated Symptoms

**Published:** 1886-10

**Authors:** T. M. Holmes

**Affiliations:** Rome, Ga.


### PHYMOSIS WITH EXAGGERATED SYMPTOMS.

BY T. M. HOLMES, M. D., ROME, GA.

Some months ago I was called to see a mulatto boy about five years of age, who had been suffering for several days from profuse diarrhœal discharges and frequent micturition. He was very much reduced in flesh; his features were pinched and his skin was dry and harsh. His eyes were of a smoky hue, and he was almost blind. He also had a papular eruption from the crown of his head to the soles of his feet, with a serous discharge not unlike that of eczema. There was more or less pain in the region of the bladder all the while, which was greatly intensified by the accumulation of a small amount of urine in this viscus. When this occurred he would run the floor, compressing his abdomen with his hands, and cry in such a manner as to enlist the sympathy of all lookers on. He had been losing his vision for several weeks and growing very nervous.

What could be the exciting cause of this amaurosis, this diseased skin and nervous phenomena? I sought to discover it, which resulted in finding an almost complete phymosis and partially closed meatus.

The diarrhœa was successfully treated, but with no abatement of the other symptoms. It was evident to my mind that the patient was suffering from a severe nervous irritability, the result of the phymosis, and a slow uræmic poisoning, the solid constituents of the urine having been re-absorbed from the retained urine in the bladder. I first sought to relieve the patient by dilatation, which failed to give the necessary relief on account of the condition of the meatus. I therefore performed circumcision, with the assistance of Mr. George Glover. The result was a complete relief and a disappearance of all the above symptoms. The boy is now in fine health.

